# Fusarium Wilt of Coriander: Root Cause Analysis and Varietal Tolerance Development

**DOI:** 10.3390/plants13152135

**Published:** 2024-08-01

**Authors:** David Chaimovitsh, Tali Kahane-Achinoam, Ohad Nuriel, Yael Meller Harel, David Silverman, Nadav Nitzan, Omer Frenkel, Itay Gonda

**Affiliations:** 1 Unit of Aromatic and Medicinal Plants, Newe Ya’ar Research Center, Volcani Institute, Ramat-Yishay 30095, Israel; davidch@volcani.agri.gov.il (D.C.); kahane@volcani.agri.gov.il (T.K.-A.); 2 Faculty of Agriculture, The Hebrew University of Jerusalem, Rehovot 7610001, Israel; ohad.nuriel@mail.huji.ac.il (O.N.); omerf@volcani.agri.gov.il (O.F.); 3 Department of Plant Pathology and Weed Science, Volcani Institute, Agricultural Research Organization, Rishon LeZion 7505101, Israel; 4 Plant Protection and Inspection Services, Ministry of Agriculture and Rural Development, Rishon LeZion 7505101, Israel; yaelm@moag.gov.il; 5 Extension Service, Ministry of Agriculture and Rural Development, Rishon LeZion 7505101, Israel; dasil@shaham.moag.gov.il; 6 Valley of Springs Research & Extension Center, Beit She’an 11710, Israel; nitzan_nadav@yahoo.com

**Keywords:** *Coriandrum sativum* L., fusarium, breeding, wilting, cilantro, tolerance

## Abstract

Since 2012, growers of coriander, *Coriandrum sativum* L., in Israel have been suffering from summer wilting that can result in entire fields collapsing. The current study aimed to determine the cause of the phenomenon and find a genetic solution to the problem. The disease was reproduced in a growth chamber using naturally-infested soil from a commercial field. Wilt became apparent within two weeks, and after ten weeks, all plants died compared to plants in sterilized soil from the same source. *Fusarium oxysporum* was isolated from infected plants, and Koch’s postulates were completed. Sequence analysis of the *Elongation Factor* (*EF1α*) encoding gene of the pathogen had a 99.54% match to *F. oxysporum* f. sp. *coriandrii*. Several coriander varieties were screened for resistance or tolerance to the disease. In four independent experiments, only the cultivar ‘Smadi’ showed high tolerance, while other genotypes were susceptible. In a trial in a naturally infested field, the cultivar ‘Smadi’ outperformed the commercial cultivar ‘Blair’. ‘Smadi’ provides a cropping solution to many Israeli farmers, yet this winter cultivar bolts early in the summer. There is a further need to characterize the tolerance mechanism and inheritance for informed breeding of late-bolting Fusarium-resistant coriander.

## 1. Introduction

Coriander, *Coriandrum sativum* L., is a leafy crop grown worldwide for culinary uses, for the cosmetic and essential oil industries, and as a medicinal plant [1]. Sometimes called cilantro, coriander is an aromatic herb of the Apiaceae family. The leaves are a popular fresh herb with a unique and distinguishable aroma, and the dry seeds are a central spice in many cuisines. Coriander is an annual plant cultivated as a summer and winter crop [2]. There is a constant demand for fresh leaves on the market, and the short shelf-life of the product [3,4] dictates year-round cultivation, which fits well with the short time needed from sowing to leaf harvest, especially in warm climates, where early-bolting limits the number of harvests [5,6,7]. On the contrary, farmers growing coriander for seed production prefer a short time to flowering, minimizing the growing period. Most seed companies indicate bolt tolerance on their coriander seed varieties since this is a critical attribute/characteristic necessary for the growers’ success. The predominant method employed in coriander breeding up to this point has been line selection. A vast collection of germplasm is available in gene banks for breeding purposes [1,8]. Recently, wild coriander accessions were suggested as a new potential genetic resource [9].

As a crop, coriander suffers from various diseases, and, until recently, stem gall and powdery mildew were the major ones causing substantial yield and income losses [10]. Several reports on Fusarium wilt caused by *Fusarium oxysporum* in coriander have recently become more frequent: the disease was initially reported in India [11], and later in Argentina [12], California [13], and Egypt [14]. The pathogen identified in India was named *F. oxysporum* f. sp. *coriandrii* [15]. The first sequencing of *F. oxysporum* f. sp. *coriandrii* was deposited for an isolate from Italy [16]. *Fusarium oxysporum* in coriander was also reported in two areas of China [17,18] and in India [19]. Lately, the genomes of two isolates of *F. oxysporum* f. sp. *coriandrii* from California have been assembled [20], and their phylogenomic relation with the *F. oxysporum* specific to celery has been studied [21]. Within celery, variation in the virulence of the different *Fusarium* isolates toward specific cultivars was reported [22]. Damping-off and the crown and root rot phenomena in coriander were also reported to be caused by *Rhizoctonia* spp. in Brazil and California [23,24]. Another soil-born pathogen reported in coriander, causing root rot and damping off, is *Pythium* spp. [25,26].

Since 2012, a wilting phenomenon has appeared in commercial Israeli coriander fields. Occasionally, entire fields undergo total collapse, leading to their abandonment. The disease is currently widespread in both open-field and netted tunnel cropping. So far, no effective chemical control has been reported for Fusarium in coriander cultivation. Yet, while open-field growers can rotate the crop to non-infested soils, those using netted tunnels are limited and need a hasty solution. We hypothesized that a fungal pathogen belonging to *F. oxysporum* species incited the phenomenon and that resistant and/or tolerant varieties may be used to control the disease. The main objectives of the present study were (1) to identify the causal agent of the phenomenon, and (2) to screen various coriander cultivars and breeding lines for resistance or tolerance.

## 2. Results

### 2.1. Symptoms and Wilting Characterization

**Figure 1:**
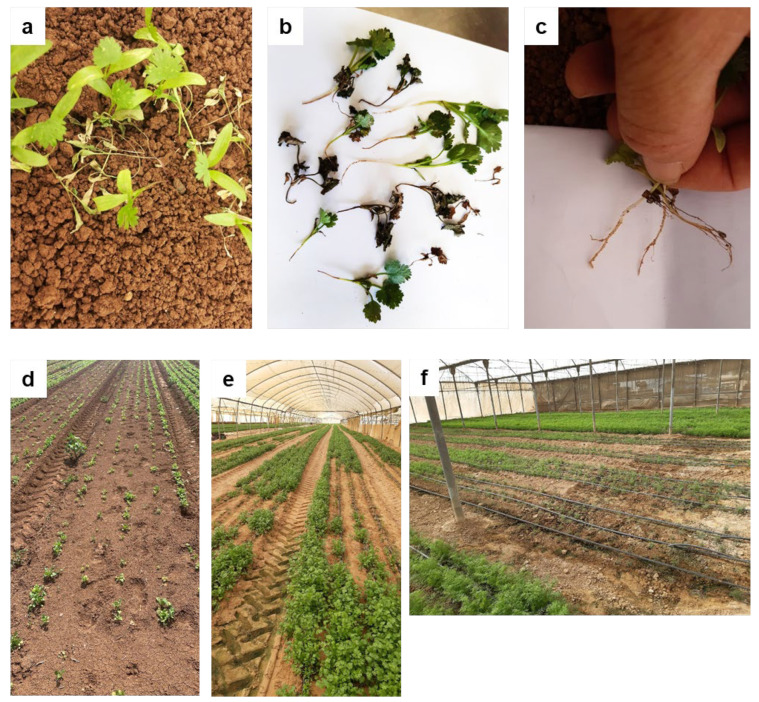
Wilting phenomenon in coriander plants. (**a**–**c**) Wilting of individual plants. (**d**–**f**) Wilting of entire fields.

The wilting of coriander has been observed in the fields in Israel during the summer season, starting approximately in 2012. Affected plants display wilting, foliar chlorosis, defoliation, and browning of the roots and crown. As symptoms progress, plants collapse and die (Figure 1a–c). The disease mainly affects young seedlings and can lead to complete yield loss (Figure 1d–f). The symptoms usually become apparent within two weeks of sowing. Since 2018, the phenomenon has become an epidemic throughout coriander cropping areas in Israel. In addition, the phenomenon is no longer limited to the summer months and can be observed as early as April, extending as late as November.

**Figure 2:**
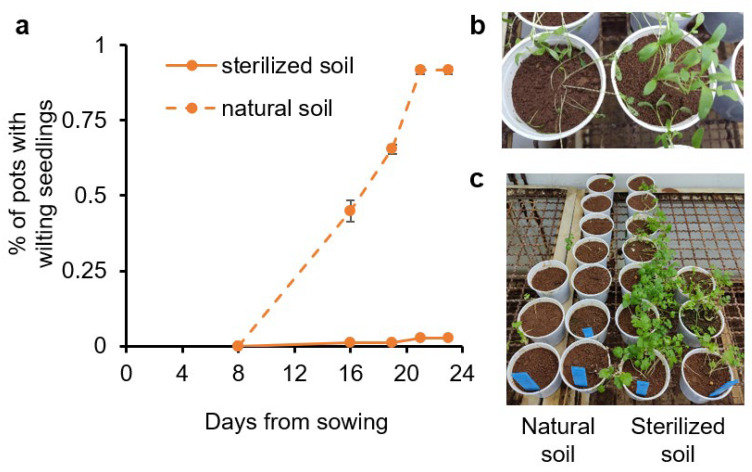
Coriander wilting in sterilized and naturally infested soil. The coriander cultivar Ya’ara was sowed in soil collected from a field with dumping-off history in a growth chamber at 27 °C. Half of the soil was sterilized (solid lines) and half was untouched (dashed lines). (**a**) The percentage of pots with wilting seedlings. The values are means of two replicates ± SEM. Each replicate contained at least 30 pots for each treatment. (**b**) Wilting phenomenon. (**c**) The final status of the experiment after 10 weeks.

To identify the etiology of the disease, naturally infested soil collected from affected fields was brought to the lab. One-half of the soil was sterilized and used as a negative control. The initial setup of the method was performed with the cultivar ‘Ya’ara’ (an early cultivar from Newe-Ya’ar) in a growth chamber at 27–29 °C with 12 h of light. About two weeks after sowing, the first wilting plants were observed (Figure 2a,b). After three weeks, all pots with non-sterilized soil displayed wilted seedlings. Ten weeks after the beginning of the experiment, almost no plants survived in the infested and non-sterilized soil, while plants in the sterilized soil were healthy (Figure 2c). A second experiment in the same design was evaluated 35 days after sowing and exhibited 91.7% ± 1.4 incidences compared to no incidences in sterilized soil.

### 2.2. Isolation of the Pathogen

**Figure 3:**
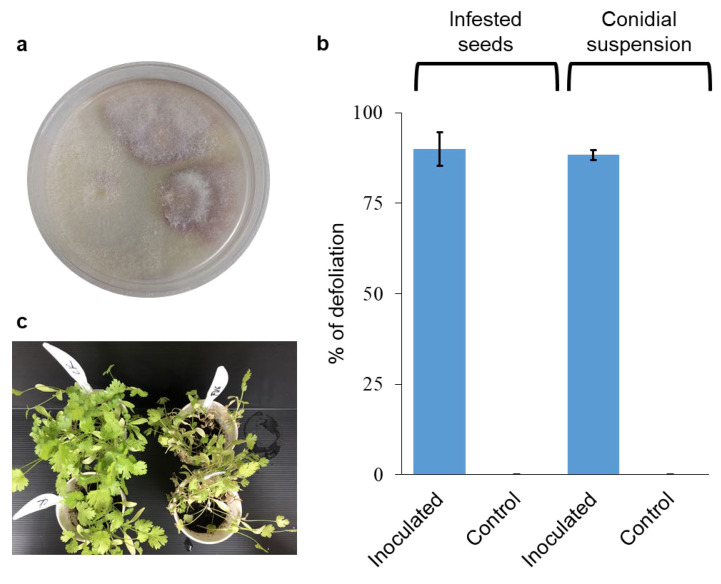
*Fusarium oxysporum* inoculation of coriander. (**a**) Isolation of *F. oxysporum* from infected plants from Magen Shaul. (**b**) Level of disease in coriander plants upon inoculation with *F. oxysporum* isolate using two different inoculation methods: infested seeds as inoculum carriers, and conidial suspension. Values are mean of 3–4 pots, respectively, ±SEM. (**c**) The status of the plants at the end of the experiment.

To identify the pathogen involved with the wilting syndrome, symptomatic plants grown on the infested soil were collected (see Material and Methods for locations). The isolates were pinkish-purple on PDA media (Figure 3a). The *Elongation Factor-1-alpha* (*EF1α*) gene was sequenced and found to be 99.54% similar (using the Clustal Omega algorithm, https://www.ebi.ac.uk/jdispatcher/msa/clustalo; accessed on 21 June 2024) to *F. oxysporum* f. sp. *coriandrii* isolated in Italy (GenBank accession number MH899124) [17], and 99.52% to the *Fusarium oxysporum* accessions isolated in China (GenBank accession number MW692008.1) [17]. The partial sequence of the *RNA polymerase II* (*RPB2*) subunit was less informative as no sequences were available in the GenBank for RPB2 from *F. oxysporum* isolates from coriander. It showed a high similarity of 99.76% to many *F. oxysporum* sequences like for instance sequences from strains associated with Asparagus (MT305137.1) and Dianthus (LT841238.1). The sequences of the two isolates, FUS3.3 and MNSE, were identical and deposited as accession numbers OR555716 and PQ082864 for the *EF1α* and the *RPB2* subunit, respectively. In two separate inoculation experiments with isolate FUS3.3, the wilting reached 90% ± 4.7 when inoculated seeds were used (method 1, see Material and Methods), and 88.3% ± 1.4 when conidial suspension (method 2, see Material and Methods) was used to inoculate the plants 15 days from sowing (Figure 3b). No wilting syndromes were observed in the non-inoculated control plants (Figure 3b,c). The *F. oxysporum* isolates were reisolated from the infected plants; hence, Koch’s postulates were fulfilled.

### 2.3. Screening for Tolerant Cultivars

#### 2.3.1. Infested Soil Experiments

**Figure 4:**
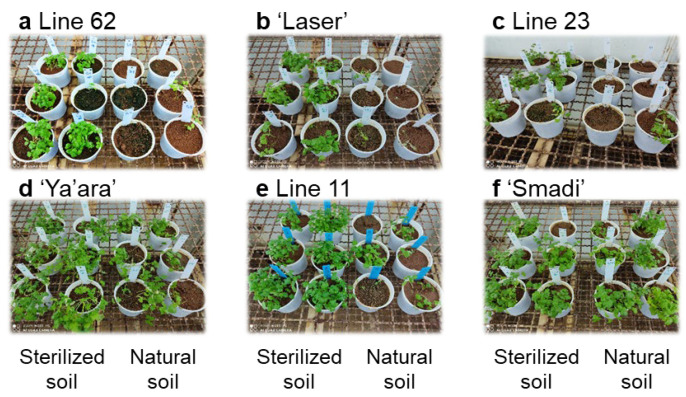
Coriander wilting in sterilized and natural soil of different cultivars and breeding lines. From each line, six pots with sterilized soil and six pots with natural soil were sowed in a completely random design in a growth chamber at 27 °C. The pictures depict the status of the plants at the end of the experiment after six weeks.

The naturally infested soil method was used to screen several coriander cultivars and breeding lines. ‘Smadi’ (a new cultivar from Newe-Ya’ar) was the only cultivar that showed a high tolerance, as was observed after six weeks (Figure 4). The three breeding lines and the commercial cultivar ‘Laser’ showed high susceptibility, while the cultivar ‘Ya’ara’ showed intermediate susceptibility in this experiment. The difference in the response of the cultivar ‘Ya’ara’ between this experiment and the initial experiments (Figure 2) is possibly due to different inoculation levels between the soils in each experiment.

**Figure 5:**
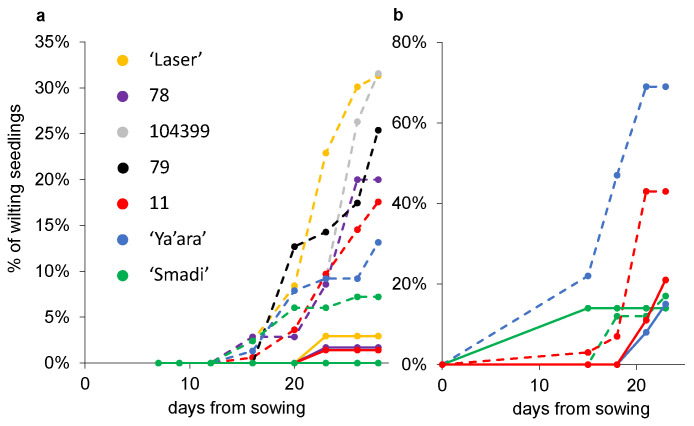
Coriander wilting progress in sterilized and natural soil. From each line, six pots with sterilized (solid lines) and natural soil (dashed lines) were sowed in a completely random design in a growth chamber at 27 °C. The percentage of the wilting plants out of the final germinated seedlings was calculated every 2–3 days. (**a**) Experiment 1. For each cultivar in each treatment, 35–161 seedlings were germinated (mean 83 ± 8.5 SEM). (**b**) Experiment 2. For each cultivar in each treatment, 13–58 seedlings were germinated (mean 28 ± 6.4 SEM).

Another two experiments were conducted where the percentages of wilted seedlings out of the total germinated seedlings were measured. In the first experiment, after 28 days, ‘Smadi’ and, to a certain level, ‘Ya’ara’, outperformed the breeding lines and the commercial cultivar ‘Laser’ (Figure 5a). Again, the first wilting seedlings were observed roughly two weeks after sowing. In the second experiment, ‘Smadi’ reached less than 20% wilted plants, similar to the results in the sterilized soil after only 23 days (Figure 5b). In this experiment, ‘Ya’ara’ reached almost 70% wilted seedlings, and line 11 reached 43% wilted seedlings.

#### 2.3.2. Artificial Inoculation

An additional variety test was conducted in large 1.7 L pots with ‘Smadi’ and four commercial varieties using an artificial inoculation with MNSE Fusarium isolate. In two experiments (using method 3, see Material and Methods), the ‘Smadi’ cultivar had significantly lower wilting rates than the other four varieties. In experiment 1, the mortality rate of ‘Smadi’ was 12.5% compared with the 40–77.5% mortality rate of the other varieties. In experiment 2, the mortality rate of ‘Smadi’ reached 40%, while the mortality rate of other varieties ranged from 72.5% to 87.5% (Table 1).

### 2.4. Field Experiment

**Figure 6:**
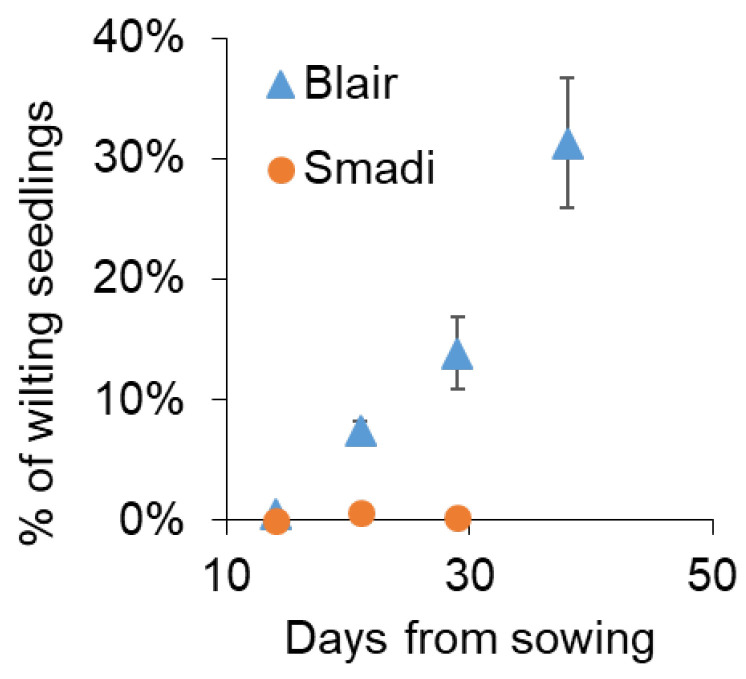
Field experiment in naturally infested soil. The cultivars ‘Smadi’ and ‘Blair’ were sown in naturally infested soil on 5 June 2023 near Magen Shaul. Percentages of wilted plants out of the germinated plants were inspected counted/determined manually in 360 cm^2^. Data shown are the mean of eight plots of each cultivar ± SEM.

To test whether ‘Smadi’ can be a commercial solution for farmers, a field trial was performed near Magen Shaul, where a history of summer wilting of coriander is common. Within 21 days from sowing, wilted plants appeared in the cultivar ‘Blair’ while ‘Smadi’ plants had minute disease levels (Figure 6). Due to its fast growth, ‘Smadi’ was harvested 30 days after sowing, and the wilting level was still negligible, while the wilting of ‘Blair’ reached 14%. The cultivar ‘Blair’ was harvested after 39 days when the wilting incidence reached approximately 30% (Figure 6). The average yield for ‘Smadi’ was 1.14 ± 0.28 (SD) kg/m^2^, and for ‘Blair’ it was 0.46 ± 0.16 (SD) kg/m^2^.

## 3. Discussion

The wilting of plants can arise from various pathogens as well as from abiotic stresses. The wilting symptoms of the coriander plants, such as chlorosis and browning of the roots and crown, pointed to biotic factors. The biotic pathogenicity of the wilted coriander in Israel was defined when soil sterilization was found to prevent the wilting. While various fungi can cause pathogenic wilting [27], *F. oxysporum* and *Rhizoctonia solani* are the most common fungal wilting agents in coriander [13,16,28]. Considering the growing evidence of *F. oxysporum* in coriander fields [25], finding it as the causative pathogenic agent in Israel was unsurprising. The distribution of *F. oxysporum* f. sp. *coriandrii* has lately been reported in the Mediterranean area. However, since the pathogenic *F. oxysporum* isolated from Israel was not 100% identical to the published *F. oxysporum* f. sp. *coriandrii EF1α* sequence, its identity as a coriander-specific pathogen has to be further studied, and more comprehensive research should be carried out to characterize the populations prevalent in Israel.

Wilting caused by pathogenic *F. oxysporum* is a common problem in many crops [29]. Currently, the status of fusarium wilt control is challenging, although sanitation and chemical or biological control have been reported to reduce the damage. Nevertheless, in most cases, only a genetic resource or transgenic plants provide disease control [30]. Usually, genetically resistant (or tolerant) cultivars persist for several years until the emergence of new and more virulent races [31]. In rare cases, resistant cultivars can remain durable for a few decades, as in the case of Fusarium-resistant basil [32,33,34]. We found that coriander is no exception. The tolerance of the cultivar ‘Smadi’ against the pathogen in various systems and methods, compared to other cultivars, might be explained by its relatively short time on the market since its release in 2021. Yet we see that ‘Smadi’ shows some wilting symptoms in some experiments, especially under high inoculum pressure. That can result from the nature of the tolerance, suggesting ‘Smadi’ is tolerant rather than resistant, with the level of inoculation determining the tolerance level. Alternatively, since ‘Smadi’ is not a single-seed descendant cultivar, a variation within the cultivar might display such a mosaical phenotype.

The current status calls for further research on the etiology and biology of *F. oxysporum* in coriander crops. That includes pathogen evolution and distribution and pathogen aggressiveness. Moreover, a fundamental study of the inheritance behavior of the tolerance ‘Smadi’ cultivar, the mechanism of the tolerance, and the genes involved will create additional tools for coriander breeders. A cross between resistant and susceptible cultivars can assist in developing molecular markers based on the recently published coriander genome [35]. Such cross-breeding can also lead directly to new resistant cultivars. This current work is pioneering in breeding *F. oxysporum*-tolerant coriander cultivars. Given the significant variation of coriander accessions and landraces, there is hope for more resistant sources to be found, as was demonstrated for the closely related celery crop [36].

## 4. Materials and Methods

### 4.1. Experiments with Naturally Infested Soil

Soil from a commercial field that suffered from reoccurring coriander wilting was collected near Magen Shaul (32.527306, 35.309750). One-half of the soil was sterilized by autoclaving (Tuttnauer, Breda, The Netherlands, model 3870ELV, https://tuttnauer.com/) for 30 min at 121 °C. The other half was kept in room conditions until used. The experiment was conducted in 0.25 L pots. Each pot was sown with 20 coriander seeds. The number of replicated pots in each experiment is indicated in the relevant figure legend. All experiments were conducted in a temperature-controlled growth chamber with 27–29 °C and 12 h of light. Plant wilt was recorded at regular intervals of 7–10 days. All commercial cultivars are available via the relevant seed companies. The ‘Smadi’ and ‘Ya’ara’ cultivars are available via the Unit of Aromatic and Medicinal Plants of Volcani Institute, Israel. Seeds of ‘Smadi’ and ‘Ya’ara’ cultivars are available commercially from the Unit of Aromatic and Medicinal Plants of the Volcani Institute.

### 4.2. Pathogen Isolation, Koch Test, and Cultivar Screening

In the fall of 2019, *Fusarium* colonies were isolated from the roots and crown of wilted coriander from a net house near Magen Shaul (32.527306, 35.309750) onto potato dextrose agar (1% *w*/*v*) amended with chloramphenicol (0.01% *w*/*v*). A monoconidial isolate coded FUS3.3 was sub-cultured onto PDA and used to complete Koch’s postulates on coriander cultivar ‘Ya’ara’ (a sensitive variety) in two trials under growth chamber conditions. The first trial was initiated in late April 2020 using infested coriander seeds (method 1). Coriander seeds were used as inoculum carriers to infest growth media (EcoTerra+ potting soil, Tuff Substrates LTD., Alon Tavor, Israel, https://tuff.co.il/) in 0.25 L plastic pots. Coriander seeds (100 g) were placed in 500 mL flasks, washed in tap water, and sterilized (30 min at 121 °C) twice, with an overnight incubation at room temperature between sterilizations. The conidia of isolate FUS3.3 were washed off a 10-day-old culture, and 5 mL of the suspension (10^6^ CFU × mL^−1^) were added to the flask and mixed. The flask was then incubated at 24 °C in the dark, and, after 14 days, the seeds were colonized by the fungus. Pots were inoculated by mixing 0.5 g of Fusarium-colonized seeds into 0.25 L of potting soil. Control pots were added with an equal weight of sterilized, but non-colonized, coriander seeds. Each pot was sown with 5 seeds of the cultivar ‘Ya’ara’ and placed in a growth chamber at 24 °C with 65% relative humidity and 12 h of light. A second trial was initiated in late May 2020 using a drenching method (method 2). Five seeds of cultivar ‘Ya’ara’ were sown into 0.25 L plastic pots in EcoTerra+ potting soil and placed in the growth chamber at 24 °C with 65% relative humidity and 12 h of light. Upon seedling emergence, the growth medium was drenched with 15 mL of conidial suspension harvested from a 10-day-old FUS3.3 culture (10^5^ CFU × mL^−1^). Control pots were added with an equal volume of sterilized distilled water. Both trials were organized in a completely randomized layout with 4 replicated pots per treatment. Disease severity was recorded 4 weeks following inoculations as a percentage (%) of foliar wilting (% defoliation).

A second *F. oxysporum* isolate, named MNSE, was isolated from wilted coriander plants in a net house near Meitar (31.317183, 34.917724) in the Negev region, about 140 km to the south of the FUS3.3 location. An inoculation test was conducted using colonized millet seeds (method 3) as described by [37]. Briefly, 40 g of millet seeds were soaked in water, transferred to 1 L flasks, and sterilized (30 min at 121 °C). MNSE isolate was sub-cultured for 120 h on potato dextrose agar (PDA) in 9-cm Petri plates and used to inoculate the millet seeds. Following 10 days of incubation at 25 °C, the Fusarium-colonized seeds were added to the potting mixture (Even-Ari Green, Moshav Beit El’azari, Israel, http://www.evenari.co.il/) at a ratio of 0.5% (*w*/*w*). Ten seeds of each coriander cultivar were sown in each pot. For each tested cultivar, five pots were inoculated, and an additional four pots served as a non-inoculated control. The pots were placed in a randomized block design, and disease incidence was documented based on plant wilting and death percentage every week.

### 4.3. Molecular Characterization

Genomic DNA from isolates FUS3.3 and MNSE (see above) were extracted by scrapping an 8-day colony grown on PDA plates. DNA was extracted with MasterPure Yeast DNA purification kit (Lucigen, https://shop.biosearchtech.com/lucigen). The isolates were identified by PCR amplifying the *EF1α* using the primers EF1/EF2 described by [38] and primers 5F2 and 7CR for amplifying part of the second largest subunit of *RPB2* [39]. The PCR product was purified by a PCR DNA Fragments Extraction kit (Geneaid, New Taipei City, Taiwan, https://www.geneaid.com/) and sequenced by Hylab (Rehovot, Israel, https://www.hylabs.co.il/).

### 4.4. Field Experiment

On 5 June 2023, a field near Magen Shual (32.531556, 35.313722) with a wilting history of coriander was sown with the cultivars ‘Smadi’ and ‘Blair’ (a sensitive commercial variety). From each cultivar, 8 plots were sown in 4 blocks. Each plot was 8 m long × 1.2 m wide. In each plot, the number of wilted plants was counted out of the total number of plants (360 cm^2^) at specific time points, and the wilt incidence was calculated.

### 4.5. Statistical Analyses

All statistical analyses were performed with JMP^®^ software v.16. Significant differences were tested using the Tukey HSD test (α = 0.05).

## Figures and Tables

**Table 1:** Mortality incidence (%) of *C. sativum* against *F. oxysporum* isolate MNSE.

	Experiment 1		Experiment 2	
Cultivar	Mortality ^1^	Tukey HSD Test ^2^	Mortality ^1^	Tukey HSD Test ^2^
‘Kruser’	42.5 + 2.5	AB	82.5 + 10.3	A
‘Laser’	40.0 + 12.2	AB	87.5 + 4.8	A
‘Santo’	77.5 + 10.3	A	87.5 + 2.5	A
‘Smadi’	12.5 + 2.5	C	40.0 + 4.1	B
‘Turbo’	55.0 + 6.4	AB	72.5 + 4.8	A
‘San Marino’	45.0 +8.6	B	n.t.	-

^1^ Values are the mean of five pots (20 seeds in a pot) ± SEM; n.t.—not tested; ^2^ Different uppercase letters represent the LSD value (least significant difference) based on Tukey HSD test (α = 0.05).

## Data Availability

The sequences of the *EF1α* and *RPB2* genes of the *F. oxysporum* f. sp. *coriandrii* isolated from Israel (FUS3.3 and MNSE), are available from NCBI under GenBank accession numbers OR555716 and PQ082864, respectively.
